# Exploring the Antifungal Potential of the Brown Macroalga *Hormophysa triquetra* : Morphological Disruptions and Structural Changes in Plant Pathogens

**DOI:** 10.1111/1758-2229.70304

**Published:** 2026-04-28

**Authors:** Shazia Bibi, Samir Jaoua, Mohammad A. Al‐Ghouti, Nabil Zouari, Mohammed H. Abu‐Dieyeh

**Affiliations:** ^1^ Department of Biological and Environmental Sciences, College of Arts and Sciences Qatar University Doha Qatar

**Keywords:** antifungal, fungicide, macroalgae, morphological alterations, seaweeds

## Abstract

Recently, macroalgal extracts have gained attention for their valuable biologically active metabolites, known for their antimicrobial activities. This study aimed at exploring the antifungal properties of the aqueous (AQ), ethanolic (ET), and methanol: chloroform (MCF) extracts of brown macroalga *Hormophysa triquetra*, collected from Ad‐Dukhan, Qatar. The extracts were evaluated for antifungal potential against *Fusarium* species, *Alternaria alternata* , and *Colletotrichum gloeosporioides*, using the agar disc method. The results revealed that the AQ extract exhibited no inhibitory action against phytopathogens, whereas ET and MCF extracts demonstrated antifungal potential against *Fusarium* species and *A. alternata.* Although fungal radial growth was not inhibited by the application of the extracts, significant morphological variations were evident indicating fungistatic behaviour of extracts. Microscopic images of the fungi treated with extracts showed hyphal deformations, compaction, abnormal thickness, vacuolization, and presence of pseudo‐hyphal morphotypes. SEM analysis further confirmed alterations in structure of the fungi treated with extracts as compared to controls. In vivo analysis on cucumber further validated the extract's ability to suppress fungal growth, with a disease incidence of 66.7% and disease severity of 37.8%. GC/MS of the extracts revealed the presence of different bioactive compounds, primarily fatty acids, in both ET and MCF extracts.

## Graphical abstract

Extracts of *Hormophysa triquetra* showed antifungal activity with marked hyphal deformities in *Fusarium oxysporum* and *Alternaria alternata* . Light microscopy and SEM confirmed structural alterations despite unchanged radial growth. Cucumber assays validated reduced disease incidence, linked to fatty‐acid–rich metabolites detected by GC–MS.

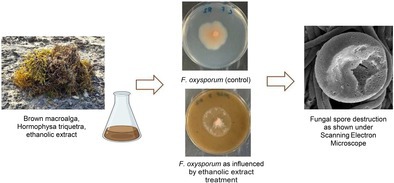

## 1 Introduction

Problems associated with phyto‐pests have increased tremendously. Although quantifying production and yield losses is challenging, estimates suggest that 20%–40% of crop production is affected or lost due to phyto‐pests (Vicente et al. 2021). Among these phyto‐pests, fungal pathogens are one of the top role players causing the most damage to crop production and yield, resulting in not only economic losses but also a reduction in the availability of food products (Savary et al. 2019). Fungal pathogens are the most aggressive disease‐causing agents, leading to 80% of plant diseases (Shuping and Eloff 2017). They are involved in pre‐ and post‐harvest crop loss and also impact the nation's trading avenues (export) that depend on cash crops (Fones et al. 2020). Fungal pathogens represent one of the most significant biotic stressors affecting agricultural productivity, with about 8000 fungal pathogens triggering crop diseases and annual crop losses (Fisher et al. 2020). Well‐known and widespread fungal phytopathogens include species from genera *Alternaria, Penicillium, Rhizopus, Geotrichum, Aspergillus, Botrytis, Cladosporium, Monilinia, Verticillium, Pythium, Fusarium*, and *Rhizoctonia* (Krylov et al. 2018; Li and Chen 2019; Tyśkiewicz et al. 2022; Saleh and Abu‐Dieyeh 2021). Species of *Alternaria*: *A. alternata* and *A. arborescens* are known for a 40% yield loss in orange plants (Aiello et al. 2020), whereas another species *A. solani* is being reported to have a yield loss of 79% in tomato plants (Adhikari et al. 2017). Moreover, species of *Fusarium* affect cereal crops like wheat, barley, cucumber, and oats globally by infecting their grains (Perincherry et al. 2019). The presence of phytopathogens leads to the death and extinction of superior crops by affecting their parts. This occurs through the release of mycotoxins that include but are not limited to aflatoxins, fumonisin, zearalenone, and patulin. Such contaminants are the major causes of economic losses specifically in agricultural fields. Moreover, once transferred to humans, mycotoxins can cause damage to the liver, kidneys, and even lead to paralysis (Makhuvele et al. 2020; Shabana et al. 2017).

Commercial agriculture relies mainly on the use of chemical fungicides to protect crops from phytopathogens. This is achieved by altering or damaging fungal cells and spores inhibition, respectively (Youssef et al. 2019). Because fungicides are cheap, accessible, and easy to apply, they are often overused without regard for their possible side effects on both health and the environment (El‐Baky and Amara 2021). Some common fungicides being used to protect crops and minimise yield losses include thiabendazole, fludioxonil, imazalil, and pyrmethanil (Chen et al. 2019). Although chemical‐based fungicides and antibiotics offer quick relief to farmers by providing immediate protection against pathogens, with time, their continued use results in the development of resistance and the bioaccumulation of chemical residues within plant systems (Pandit et al. 2022). Generally, the biodegradability of fungicides is low; hence, high accumulation within the environment (Santra and Banerjee 2020). For instance, the isolation of strains of *Alternaria* from pistachio fields in California, sprayed with fludioxonil, has confirmed their resistance to the fungicide over time (Avenot and Michailides 2015). With heavy reliance of farmers on fungicides with limited spectrum, it has become challenging to cope with the growing phytopathogens, most with developed resistance towards the already available fungicides. The biological method of controlling pathogens relies on non‐chemical techniques involving the use of biological entities for disease management and biocontrol. These methods are preferred over chemical methods because they are safe for both living beings and the environment. Different extracts, natural and essential oils, resins, and other such compounds are being extracted from biological bodies to be used as efficient antimicrobial agents (Kokoska et al. 2019). Not only are plant materials used for extraction purposes, but microbes are also exploited for disease management in crops (Ayaz et al. 2023). Traditionally, extracts from plants are highly preferred when it comes to the assessment of antimicrobial activities. One of the main reasons is the presence of a versatile group of bioactive compounds in plant extracts, including—but not limited to—phenolics (Gatto et al. 2011), flavonoids (Dias et al. 2021), coumarins (Sharifi‐Rad et al. 2021), catechin (Bae et al. 2020), and tannins (Das et al. 2020).

Seaweeds are home to significant bioactive compounds with profound functions, such as phenolic compounds, fatty acids, terpenes, sterols, and other related groups (Milledge et al. 2016; Rizzo et al. 2017; Akbar and Mustari 2024). These compounds have plant growth‐promoting properties due to the presence of bio‐stimulating components (Kapoore et al. 2021; Akbar and Hasan 2024). Moreover, the macroalgal extracts have antimicrobial properties and are known to inhibit the growth of different plant pathogenic fungi. Naturally existing compounds in macroalgae are considered an alternative and are being tested for their antifungal potentialities through in vitro and/or in vivo methodologies. In vitro assessment mainly relies on the study of either mycelial inhibition or inhibition at the sporulation level (Taskeen‐Un‐Nisa et al. 2011). Literature reports the use of all three types of macroalgae in combating against phytopathogens. For example, red macroalga *Gracilariopsis persica* has been reported to inhibit the mycelial growth of different phytopathogens that include *Botrytis cinerea, Aspergillus niger*, and *Pyricularia oryzae* (Pourakbar et al. 2021). Crude extracts of brown macroalgae *Sargassum muticum* , *Padina gymnospora* , and *S. wightii* are utilised against *Rhizoctonia solani*, known to cause sheath blight disease in rice (Raj et al. 2016). Similarly, another study reported that spraying extracts of macroalga *Ascophyllum nodosum* reduced leaf blight disease in carrots caused by *A. radicina* and *B. cinerea* (Jayaraj et al. 2008). A brown macroalga *Bifurcaria bifurcate* showed strong efficiency in inhibiting the mycelial growth of species of *Penicillium* (Fayzi et al. 2022).

Brown macroalgae, in general, are valued more over red macroalgae due to their metabolite profile. They have a plethora of lipophilic compounds that include both saturated and unsaturated fatty acids (Biris‐Dorhoi et al. 2020). Furthermore, the presence of different phenolic compounds/polyphenols makes them a strong candidate for use as a biocontrol agent (Cowan Marjorie 1999). Among the three macroalgal groups, brown macroalgae are rich in polysaccharides that are also indicative of strong antimicrobial and other biological properties. Due to the presence of such a versatile group of compounds, brown macroalgae are reported to exhibit stronger antimicrobial activity compared to green and red macroalgae (Natrah et al. 2015). Multiple studies have reported antifungal activity of brown macroalgae through GC–MS profiling; but these investigations were directed towards fungistatic outcomes, pot‐based disease suppression experiments, gene‐based analysis, or antioxidative profiling, without examining direct pathogen‐extract interactions through structural or morphological alterations. In many studies, structural evidence linking bioactive metabolites of extracts to fungal disruptions was lacking. Moreover, macroalga *Hormophysa triquetra* has received comparatively less attention despite its valuable chemical composition. In the present study, GC–MS profiling along with SEM‐based structural disruptions and fruit infection assays enabled us to present a hypothesis‐based interpretation of antifungal activity of macroalgal extracts at the pathogen structural level.

The Arabian Gulf is a semi‐enclosed sea that connects the Arabian Sea through the Sea of Oman and the Strait of Hormuz. The Arabian Gulf has hot summer, high evaporation rates, low precipitation, moderate winters, high winds, and dusty storms (Aboobacker et al. 2021). The Gulf is popular for it being hypersaline naturally (Rakib et al. 2021). Qatar being surrounded by many islands has typically hot and saline seawater which is home to different organisms including macroalgae (Al‐Ashwal et al. 2020). So far, it has been reported that there are 67 macroalgal species in the islands surrounding Qatar (Al‐Ashwal et al. 2020). The goal of this study was to analyse the antifungal activity of extracts of brown macroalga *H. triquetra* collected from the Qatari coast. *H. triquetra* was selected for this study due to its abundance and availability throughout the year as the specie is not seasonal. The collection of this macroalga is easier compared to others due to its larger size.

## 2 Materials and Methods

### 2.1 Sample Collection

*H. triquetra* (HT) was sampled manually between high and low tides from Ad‐Dukhan (25°30′10.8″ N 50°50′05.4″ E), Qatar in June 2023. The samples were cleaned with seawater to remove debris and other unwanted macroalgae. The samples were transported to the lab in thermocol boxes (Bibi et al. 2025).

### 2.2 Sample and Extract Preparation

In the laboratory, the macroalgal samples were washed thrice with Milli‐Q water. The samples were kept in a shady area to be air‐dried (Kumar et al. 2013). The air‐dried samples were then ground to powder and stored at 4°C. Three extracts, namely Aqueous (AQ), ethanolic (ET), and methanol: chloroform (MCF), were prepared from the macroalgal samples. For the preparation of AQ extract, 30 g of powder was added to 300 mL sterile distilled water in a sterile flask and was incubated in a water bath set at 60°C for 20 min. The mixture was stirred constantly. For the ET extract, the powder was added to 70% ethanol in a similar ratio as described for the preparation of AQ extract. The mixture was incubated in a shaker at 35°C for 24 h at 120 rpm. The MCF extract was prepared by the addition of 30 g of powder to a 300 mL 1:2 ratio methanol and chloroform solution. The mixture was incubated in a shaker at 35°C for 24 h at 120 rpm. For all the extracts, after completion of incubation time, the mixtures were filtered using Whatman filter paper. The filtrates were then added to sterile glass plates and kept for drying in a preheated oven at 40°C (Bibi et al. 2025). The extracts were prepared in triplicates to ensure the consistency of yield.

The extraction yield was calculated based on the Equation (1) given below (Dhanani et al. 2017) and was represented along with standard deviation (SD):

(1) $$\text{Extraction yield}\;\left(\%\right)=\frac{\text{mass of extracted powder}}{\text{mass of biomass powder}\;}\times 100\%$$

### 2.3 In Vitro Assessment of Antifungal Activity of Extracts

The antifungal activity of the three prepared extracts was tested against *A. alternata, C. gloeosporioides, F. oxysporum* (PV226192), and *F. oxysporum* 2 (PV247065) in triplicates. These species were isolated previously from spoiling food and molecularly identified (Saleh and Abu‐Dieyeh 2021). The agar diffusion method was employed to test the efficacy of the extracts against the selected fungi. Three different extract concentrations, 1, 5, and 20 mg/mL were used to evaluate the antifungal activities of the extracts. The extracts were prepared by dissolving the extracted powder in distilled water and then autoclaving at 121°C for 15 min (Escobedo et al. 2020). Extracts' antifungal activity improved post autoclaving. Following autoclave, the extracts were subjected to sonication for 10 min.

#### 2.3.1 Agar Diffusion Method

Potato dextrose agar (PDA) with desired concentrations of the three extracts (1, 5, and 20 mg/mL) was prepared by adding the calculated volumes from stock solutions (40 mg/mL) of extracts. The extracts were added to the autoclaved PDA with a temperature maintained at 50°C. The stock concentrations of extracts were prepared by the addition of desired extract powder to sterile distilled water (Saleh and Abu‐Dieyeh 2021). The PDA was set to solidify after which a disc of 6 mm was removed under sterile conditions from the centre of the solidified plates. Fungal discs of 6 mm size from the margins of 4‐day fungal cultures were removed and added to the PDA plates at the emptied centre. For positive control, 1 mL Clotrimazole was used per plate with a concentration of 10 mg/mL. Triplicate cultures were incubated at 25°C for 5 days. The radial growth of fungi was calculated by measuring the colony diameter in two perpendicular directions. The calculations were done with the help of the following Equation (2) (Hendricks et al. 2017):

Percent radial growth inhibition (RGI %) = (*d*_*c*_−*d*_*t*_) × 100/*d*_*c*_ where *d*_*c*_ and *d*_*t*_ represent the diameter of the fungal colony in control and treatment plates.

(2) $$\text{Radial growth inhibition}\;\left(\mathrm{RGI}\%\right)=\frac{\text{diameter of the fungal colony in control}-\text{diameter of the fungal colony in treatment}}{\text{diameter of the fungal colony in control}}\;\times \;100$$

The fungal morphology was studied by microscopy using the ZEISS Axiostar plus microscope, Carl Zeiss Microscopy, Germany. Fungi from the margins of their fresh culture plates of both control and treatment groups were taken and added to a drop of cotton blue on a glass slide. The slide was covered with a cover slip and then observed under the microscope at a magnification of 40 X. Photomicrographs were captured with a mobile camera positioned over the eyepiece. The effect of the extract on fungal morphology was evaluated based on established criteria, including alterations in hyphal structure, degree of septation, branching pattern, sporulation, and spore size. To assess the effect of contact time between the fungi and the extract, the spores of fungi were added to the extracts at different concentrations and incubated for different time periods of 15, 30, 60, 90 min, and overnight. A disc of approximately 6 mm was plunged from PDA plates alone and PDA plates supplemented with extracts. To the hollow centres, 10 μL of spores and extract mixture were added and incubated at 25°C for 5°C days. To verify the results, extracts using the same methodology and conditions were prepared and tested independently.

#### 2.3.2 Investigation of the Effect of Extracts on Fungi by SEM Analysis

To further validate the effect of ET and MCF extracts, SEM analysis was used. The fungi were allowed to grow on plates with extracts, while for control, the fungi were grown on normal PDA plates. The plates were incubated at 25°C for 5 to 7 days until the desired results were obtained. This method enabled us to study the growth of fungi and to observe its hyphae and spores. Further, the mechanism of action of extract against phytopathogenic fungi was also investigated through SEM analysis. From the PDA plate of both control and treatment groups, the mycelia of fungi were obtained (Alves et al. 2013) and transferred to an eppendorf tube with the fixative solution (2.5% glutaraldehyde and 2.5% formalin). The samples were incubated (at least 24 h) in the refrigerator until the next step. The samples were then washed with phosphate buffer saline (PBS) thrice. The samples were then post‐fixed in a 1% osmium tetroxide solution and incubated for 1–4 h at room temperature. The samples were then washed with distilled water thrice. Lastly, the samples were dehydrated in a series of ethanol solutions (25%, 50%, 75%, 90%, and 100%) with an incubation of 10 min in each step. The samples were then smeared and gold coated on metallic studs to be observed under Nova NanoSEM450 at different magnifications: 2500 × (scale: 50 μm), 5000 × (scale: 50 μm), and 10,000 × (scale: 10 μm) (Bibi et al. 2025). The same morphological evaluation criteria were used to assess the effects of extracts on fungi. ImageJ software 1.54p by National Institutes of Health, USA (https://imagej.net/software/fiji/) with scale set to μm was used to measure the features of fungi. Quantitative shape descriptors like area, aspect ratio (AR, values close to 1 indicate symmetry, and above 1 indicates asymmetrical structure), circularity (close to 1 indicates regular circle), roundness (0–1), and solidity (smoothness and compaction of structures) were measured to characterise the spore structural uniformity in control and treatment groups.

### 2.4 Fruit Bioassay to Assess Antifungal Activity of Extract Against *F. oxysporum* (PV226192) on Cucumber

Cucumbers (Mahaseel Premium, Hassad Food, Qatar) with no visible decay or infection were purchased from a local store in Qatar. To assess the antifungal activity of the macroalgal extracts in vivo, a curative experiment was conducted. Three replicate batches were used for the experiment. Each replicate had five cucumbers. Three pricks were made in cucumbers using a sterile needle. The wounds were air‐dried after which they were inoculated with 20 μL of spore suspension of *F. oxysporum* (PV226192) (10^6^ spores/ml). After inoculation of spores, the wounds were left to dry again and then sprayed twice with 5 mg/mL of ET extract (Saleh et al. 2022). Between each spray, the wounds were allowed to air‐dry. The selection of extract was done based on the most morphological alterations it has caused in the tested fungal pathogens. All the cucumbers were stored in sterile plastic bags in an incubator with a temperature of 25°C and humidity of 75%. Cucumbers were observed for changes for 7 days after which disease incidence (DI), disease severity (DS), and percent extract efficacy (% EE) were measured (Xiao et al. 2018). Disease symptom evaluation was not performed blindly; however, assessments were conducted using a standardised method by the same evaluator to validate consistency. The following equations were used to measure the mentioned parameters:

(3) $$\mathrm{DI}=\frac{\text{Number of rotten cucumbers}}{\text{Total number of cucumbers}}\times \;100\%$$

(4) $$\mathrm{DS}=\frac{\text{Average lesion diameter of treated cucumbers}}{\text{Average lesion diameter of control cucumbers}}\;\times \;100\%$$

(5) $$\mathrm{EE}\;\left(\%\right)=\frac{\left(\text{Disease incidence in Control group}-\text{Disease incidence in treatment group}\right)}{\text{Disease incidence in Control group}}\;\times \;100\%$$

Two‐tailed student's *t*‐test was performed in Sigma Plot (version 15) at *p*‐value < 0.05 to evaluate the significance of results.

### 2.5 GC/MS Analysis of Extracts

The GC/MS was performed with a GC system 7890A‐5973 network mass‐selector detector (Agilent, Santa Clara, CA, USA) in MS scan mode between 45 and 500 m/z mass range. Helium was used as a carrier gas (Flow rate: 1.50 mL/min). Approximately 1 μL of sample was injected into the Rxi‐5Sil MS GC column with size 30 m, 0.32 mm ID, 0.25 μm in split ratio. The column temperature was maintained at 80°C and then increased to 250°C (15°C/min). The compounds were identified by comparison with the NIST 20 library.

## 3 Results and Discussion

### 3.1 Extraction Yield (%)

Based on the preliminary antifungal activities of the extracts, the study proceeded with only ET and MCF extracts. However, the extraction yield (%) for AQ is still reported for research purposes. The extraction yields for the three prepared extracts were calculated based on Equation (1) and are presented in Table 1.

**TABLE 1:** Extraction yield (%) *±* Standard deviation (SD) of Aqueous (AQ), ethanolic (ET), and methanol: Chloroform (MCF) extracts.

Extract	Average extraction yield (%) ± SD
AQ	16.35 ± 1.35
ET	20.50 ± 2.05
MCF	6.43 ± 1.16

The extract yield varies by species, season, and location (El‐Sheekh et al. 2023). Further, the type of extraction method utilised, considering the time, temperature, and solvent used, is also another factor affecting the extraction yield. Three different types of solvents are utilised in this research to extract bioactive compounds from macroalgae. Water (AQ) solvent with a polarity index of 9, is highly polar and is mainly used to extract bioactive compounds such as amino acids, saponins, sugars, lectins, anthocyanins, polypeptides, and starches. Ethanol (ET) has a medium polarity with a polarity index of 5.2 and can extract other polar bioactive compounds like tannins, flavanols, polyphenols, polyacetylenes, propolis, sterols, anthocyanins, alkaloids, and terpenoids (Azmir et al. 2013; Moulishankar et al. 2021). MCF, on the other hand, is a mixed solution of methanol (polarity index: 6.6) and chloroform (polarity index: 4.1) used to extract neutral to polar lipids (Axelsson and Gentili 2014; Zarrinmehr et al. 2022). This combination (2:1 v/v) is reported to be the best in terms of having a higher yield of lipids (Saini et al. 2021). In the combination of this solvent, methanol behaves as a disrupter that is able to break the strong bonds between lipids and proteins, whereas the other counterpart, chloroform acts as a mediator that accelerates processes such as diffusion and extraction of lipids from the initial biomass (Bibi et al. 2025). Brown macroalgae, in general, is composed of a variety of bioactive metabolites ranging between highly polar to moderately polar, and to low‐ or non‐polar compounds. It was essential to select solvents that could extract a wide range of bioactive compounds.

In literature, limited information is available on the extraction yield from macroalgae utilising different solvents. However, some studies reported the ethanolic extraction yield for *Asparagopsis armata* , *Codium* sp., *Fucus vesiculosus* , and *S. muticum* to be 2.01%, 5.96%, 8.60%, and 1.22%, whereas the extraction yield of water as a solvent was 7.91%, 49.78%, 28.69%, and 8.99% respectively (Toledo et al. 2023). In our study, the ET extraction yield is much higher than the reported ones, whereas the AQ extraction yield lies within the mentioned range. For lipid extraction, methanol and chloroform have been used individually and in combination, as reported in different studies. For instance, methanol, chloroform, and a mixture of both solvents were used to extract lipids from *Cladophora glomerata* with extraction yields of 8.6%, 7.6%, and 9.5% (Yuvarani et al. 2017). Our extraction yield, with methanol and chloroform solvent, was 6.43%, which is somewhat close to the reported values. Extracts were subjected to sterilisation by autoclaving it, followed by sonication prior to evaluating their antifungal potentialities. In our study, we found that autoclaving improved the antifungal properties of extracts. This could be due to an increase in flavonoids or phenols concentration in the autoclaved extract as heat promotes the release of alcoholic compounds. Heat not only breaks cell walls and other cellular components but also dissociates conjugated polyphenols to smaller and simpler phenolic compounds (Beta and Hwang 2018). It is reported that the extracts of rhizomes of dragon's blood and the fruit of xoconostle showed no difference in antibacterial activity against *Streptococcus mutans* and both types were able to inhibit its growth. Moreover, a lower concentration of extract was able to inhibit growth of *S. mutans* after autoclaving compared with non‐autoclave extract identified by measuring MIC (Terrazas‐Hernández et al. 2018). There are possible side effects of autoclaving extracts such as modification of bioactive compounds. Certain compounds are not thermally stable and lose their antioxidative, antimicrobial or other such properties once exposed to high autoclaving temperatures (Yin et al. 2019).

### 3.2 In Vitro Assessment of Antifungal Activity Against Phytopathogens

#### 3.2.1 Agar Diffusion Method

**FIGURE 1:**
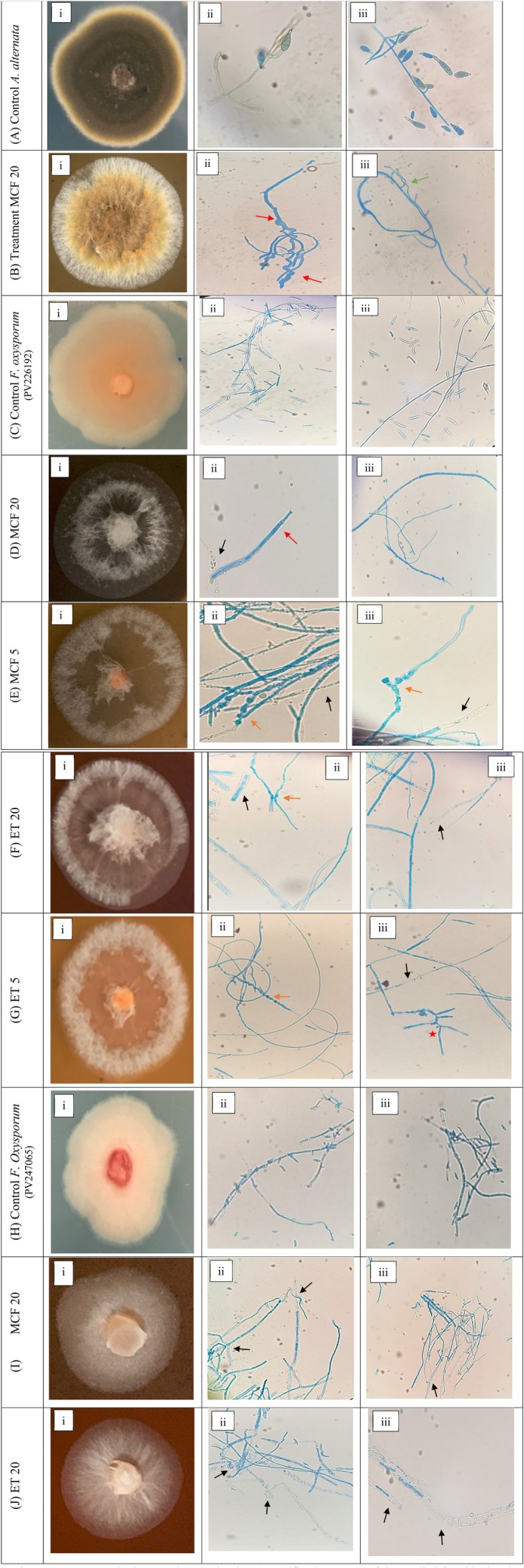
Compound microscopic examination (magnification 40×) of the studied fungi isolated and treated with different extracts. (A) Control untreated *A. alternata* ; (B) *A. alternata* treated with methanol: Chloroform (MCF) 20 mg/mL; (C) Control untreated *F. oxysporum* (PV226192); (D) *F. oxysporum* (PV226192) treated with methanol: Chloroform (MCF) 20 mg/mL; (E) *F. oxysporum* (PV226192) treated with methanol: Chloforom (MCF) 5 mg/mL; (F) *F. oxysporum* (PV226192) treated with ethanolic (ET) 20 mg/mL; (G) *F. oxysporum* (PV226192) treated with ethanolic (ET) 5 mg/mL; (H) Untreated *control F. oxysporum* (PV247065); (I) *F. oxysporum* (PV247065) treated with methanol: Chloform (MCF) 20 mg/mL; and (J) *F. oxysporum* (PV247065) treated with ethanolic (ET) 20 mg/mL. Green arrow: Pseudo‐hyphae morphotype; Red arrows: Thick deformed hyphae; Black arrows; disintegrated and collapsed hyphae; Orange arrows: Vacuolization of hyphae; Red star: Hyphal anastomosis.

The AQ showed no to least inhibition against the tested fungal strains (results not shown); therefore, the experiments were continued with ET and MCF only. The radial growth of the fungi was not inhibited by the extracts ET and MCF, as shown in Table 2, however, morphological differences were observed for the reported species (Figure 1). For *C. gloeosporioides*, neither radial growth was inhibited nor any morphological changes were seen. For *A. alternata* treated with ET, the radial growth was inhibited by 14%, 10.11%, and 8.91% at concentrations of 1, 5, and 20 mg/mL. For *F. oxysporum* (PV226192), the radial growth inhibition at 1 and 5 mg/mL were 9.52% and 13.01%, respectively; however, at 20 mg/mL, the fungus grew bigger than the control by 2.53%. A similar trend was observed for *F. oxysporum* (PV247065) at a concentration of 5 mg/mL, where the fungi had a bigger colony than the control by 7.51%. Percent inhibition at 1 and 20 mg/mL were 16.20% and 3.24%, respectively. MCF extract inhibited *A. alternata* at 1, 5, and 20 mg/mL by 2.38%, 9.52%, and 14.29%. For the strain of *F. oxysporum*, the colony grew bigger than the control by 3.17%, 5.07%, and 12.37% at 1, 5, and 20 mg/mL. The radial growth of the second strain of *F. oxysporum* (PV247065) was inhibited by 8.69%, 2.74%, and 28.45% at 1, 5, and 20 mg/mL, respectively. In the context of measuring radial growth, the maximum growth of fungi was measured; however, the growth in the middle was disrupted, creating empty spaces indicating weak/still growth or dead mycelia (Figure 1D).

**TABLE 2:** Radial growth inhibition with standard deviation (SD, *n* = 3) of *A. alternata, F. oxysporum* (PV226192), and *F. oxysporum* (PV247065) by ethanolic (ET) and methanol: Chloroform (MCF) extracts at concentrations of 1, 5, and 20 mg/mL.

	Species	1 mg/mL	SD	5 mg/mL	SD	20 mg/mL	SD
ET	*A. alternata*	14	0.52	10.1	2.58	8.9	1.79
	*F. oxysporum* (PV226192)	9.5	4.37	13.0	2.75	−2.5	1.46
	*F. oxysporum* (PV247065)	16.2	0.69	−7.5	1.17	3.2	3.73
	*C. gloeosporioides*	9	1.56	9.5	2.70	3.2	6.04
MCF	*A. alternata*	2.4	1.03	9.5	1.03	14.3	0.89
	*F. oxysporum* (PV226192)	˗ 3.2	2.40	−5.1	1.98	−12.4	3.44
	*F. oxysporum* (PV247065)	8.7	2.38	4.7	1.37	28.5	0.68
	*C. gloeosporioides*	0.9	3.90	4.5	6.24	−5	0.78

Radial growth stimulation of *F. oxysporum* (PV226192) and *F. oxysporum* (PV247065) was observed when treated with ET extract concentrations of 5 and 20 mg/mL. Similarly, MCF extract at all the tested concentrations stimulated radial growth of *F. oxysporum* (PV226192) and *C. gloeosporioides* at 20 mg/mL. Fungi utilise extracts that are nutrient rich as a source of food to grow (Saleem and Ali 2017). Generally, fungi produce many enzymes that transform and stabilise different compounds. Due to higher carbon to nitrogen ratio in the fungal biomass, carbon utilisation efficacy of fungi is enhanced (Karhu et al. 2022). Though biological extracts are associated with microbi‐toxic or microbicidal nature, these have growth enhancing properties for fungi and are chemically proved to be nutrient rich. Glycosides or carbohydrates (reducing sugars) are found in abundance in the extracts providing a good source of food for fungi. These glycosides are rich in glucose and a non‐carbohydrate counterpart called genin or aglycone that are organic compounds (Karhu et al. 2022).

The results of the antifungal experiments showed that the most efficient among the three extracts was the MCF and ET extracts based on the induced morphological alterations observed visually. Both extracts showed efficacy against the tested fungal strains except *C. gloeosporioides*. The microscopic examination showed evident morphological differences between the control and the treatment plates. The contact times between fungi and the extract did not show any significant differences between the results obtained from directly transferring (with no contact time between extract and spores) the fungal disc in a PDA plate supplemented with extracts.

For *A. alternata* , the extracts of ET and MCF at all concentrations showed changes in the colony morphology when compared with the control, where it was sporulating under normal conditions (Figure 1A(i–iii)). In the presence of extract, the fungus grew abnormally. By appearance, the colony was very different from the control colony, with an empty centre and whitish hair‐like structures at the margins (Figure 1B(i)). The light microscopic examination of the plate showed that in the presence of MCF at 20 mg/mL, the sporulation phenomenon of the fungus was affected along with deformation in its morphology as indicated by red arrows (Figure 1B(ii)) (Xie et al. 2021). The presence of extract caused abnormal thickness of the mycelium along with excessive coiling and elongation. Moreover, in the treatment plate, a pseudo‐hyphae morphotype could also be seen marked with a green arrow (Figure 1B(iii)) (Francisco et al. 2019). This effect was observed even at low concentrations of 1 and 5 mg/mL.

Extracts of ET and MCF at all concentrations showed effectiveness against *F. oxysporum* (PV226192). Figure 1C(i–iii) shows a normally growing *F. oxysporum* (PV226192) colony alongside its light microscopic images. On the other hand, the fungus, in the presence of extract, indicated abnormal growth. The hyphae grew from centre to margins, with empty spaces seen in between, demonstrating dead hyphae or mycelial cells (Figure 1D–F(i)). Further, disrupted and thickened mycelial growth was seen in plates treated with MCF 20 mg/mL. Black arrows indicated the disintegration and collapse of hyphae (Figure 1D(ii)), while thick and thin hyphae can be observed as well (Figure 1D(iii)). The lower concentration of 5 mg/mL of extract appeared to affect the fungus differently by compaction of mycelia, enhancing vacuolization (indicated by the orange arrow), and inhibition of sporulation (Figure 1E(ii,iii)) (Samsatly et al. 2016). Similar events were observed under the influence of ET 20 mg/mL, where dense vacuolization, damaged, disintegrated, and abnormally thickened hyphae were visible (Figure 1F(ii,iii)). Under a low ET concentration of 5 mg/mL, compacted hyphae indicated by the orange arrow (Figure 1(G(ii))), and the process of hyphal anastomoses was observed, as marked by a red star (Figure 1G(iii)), both corresponding to the visually deformed fungal plate (Figure 1G(i)). For the second strain of *F. oxysporum* (PV247065), changes in the growth of fungi were very evident in higher concentrations of 20 mg/mL for both ET and MCF (Figure 1I,J) when compared with its control (Figure 1H(i–iii)). Figure 1I(i) shows a morphologically altered fungal plate with coiled hyphae of varying width (Figure 1I(ii,iii)). Figure 1J(i) shows stressed growth of fungi with thick and collapsed hyphae (Figure 1J(ii,iii)). For the lower concentrations of 1 and 5 mg/mL of ET and MCF, fungal growth appeared to be less stressed, with very little to no differences in colony formation (results not shown).

The present study highlighted the antifungal activity of two extracts ET and MCF of brown macroalga, however, AQ extract showed no antifungal activity. The inability of AQ extract to inhibit fungal growth might be due to the limited extracting capacity of solvent as well as the concentration of active components in the extract. Generally, active ingredients are soluble in alcoholic solvents than water (Kalidindi et al. 2015). Moreover, the unlikelihood of extracting hydrophobic compounds in AQ extract could be one of the possible reasons to not have antifungal activity. Hydrophobic components in extracts are likely to interact with lipid membrane of fungi leading to membrane disruptions and associated morphological damages. The species treated with extract showed differences in the fungal morphology though the radial growth was not affected much. This phenomenon has been described in the literature as minimum effective concentration (MEC), which is the lowest concentration of drug required to cause morphological changes (Kurtz et al. 1994; Soares et al. 2016; Silva et al. 2018). Reduced and abnormal mycelial growth, no/low sporulation, coagulated cytoplasm, coiling and compaction of hyphae, and dense vacuolization were all seen under a light microscope for PDA plates with extracts. All these characteristics define the antifungal capability of the extracts against phytopathogens (Farbo et al. 2018). Similar results were reported by Silva and his team (2018), where the extracts of macroalga *Osmundea pinnatifida* were tested against fungi *Aspergillus fumigatus* and *A. infectoria (*Silva et al. 2018). The study reported that the extracts affected the process of conidiation along with morphological alterations. Similarly, in another study, the extracts from macroalga *Chondracanthus teedei* var. *lusitanicus* were tested against *A. fumigatus* and *A. infectoria*. The results showed the formation of hyphal segments that were swelled, highly branched, and shorter in size (Soares et al. 2016). Aqueous extract of *Undaria pinnatifida* affected the fungus *Phytophthora infestans* morphologically by altering the hyphal diameter, branching, and reduction in spores number (Lobato et al. 2023). Previously, such alterations in the morphology of fungi were seen with the application of volatile organic compounds (VOC's). For instance, the effect of VOCs produced by *Chromobacterium vaccinii* on fungi *Coleophoma* sp., *Phoma* sp., *Colletotrichum* sp., and *Phytophthora cinnamomi* resulted in their hyphal membranes to be pulled off from the cell wall, leaked hyphal bodies, abnormally swelled and deformed hyphae, and lack of sporangia development (Ebadzadsahrai et al. 2020). Another study, where the VOCs released by *Lachancea thermotolerans* 751 coagulated the hyphal cytoplasm of *A. ochraceus* . Further, it caused the hyphal tips to swell, leading to hyphal lysis (Farbo et al. 2018). The reported mechanisms through which extracts inhibit the fungal growth is by disrupting fungal membranes. The extracts initiates disruption of membranes which further disrupts the electron transport chain (ETC), leading to an increase in fluidity of membrane. These changes cause conformational disorders, expressed through leakage of components from cytoplasm eventually resulting in death of fungi (Mohamed and Saber 2019). Furthermore, it is suggested that the concentration of chitin and glucan, the two major components of the fungal cell wall decreases due to application of extracts, leading to the deformation of fungal hyphae. This could also be supported by the fact that the antifungal drugs are designed to attack chitin and glucan (Munro 2013; Jacobs et al. 2021). Other potential mechanisms utilised by extracts to inhibit fungal growth is through penetration in membrane and interacting with nucleic acids or mitochondria. This interaction with nucleic acids disrupts or inhibits synthesis of proteins leading to instability within the cell (Lopes et al. 2013). Fungal membrane is composed of sterol responsible for membrane structure, rigidity, permeability, and proteins' activity (Eliaš et al. 2024). Some compounds found in extracts are able to inhibit or interact with synthesis of sterol, disturbing normal regulation of membrane modulators and increasing membrane fluidity (Gutierrez‐Perez and Cramer 2025). Structural and morphological deformations of fungi observed by SEM in the absence of radial growth inhibition is indicative of a fungistatic effect rather than reduced antifungal efficacy. Stress‐induced morphogenesis observed is a recognised feature of fungal response to membrane‐linked stress. (Tenorio‐Salgado et al. 2013; Kai et al. 2013). The fungistatic nature of macroalgal extracts are beneficial in agriculture as it will not effect the overall fungal biodiversity present under natural conditions in field rather than inhibiting their growth as described by Sepulveda and team (2024) for fungistatic effects of *Capsicum* pepper extracts against phytopathogens (Sepúlveda et al. 2024).

### 3.3 Investigation of Mechanism of Action of Extracts Against Phytopathogen by SEM Analysis

**FIGURE 2:**
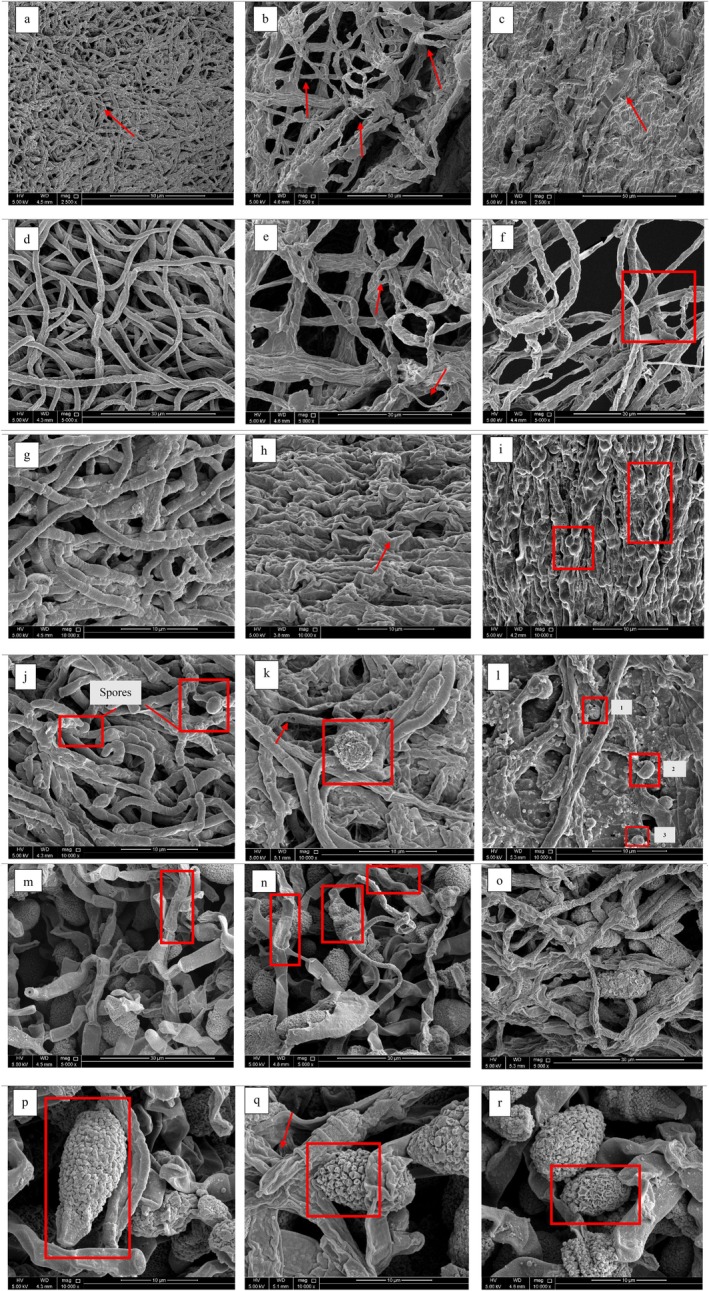
SEM micrographs of: *F. oxysporum* (PV226192) untreated (A, D, and G), treated (B, C, E, F, H, I), *F. oxysporum* (PV247065) untreated (J), treated (K and L), *A. alternata* untreated (M and P), treated (N, O, Q, R). Magnification and scale (2500×, 50 μm (A–C)), (5000×, 50 μm (D–F, M–O)), (10,000×, 10 μm (G–L,P–R)).

To further validate the results, the mechanism was studied by SEM analysis. The SEM images of the control and treatment plates showed evident differences in morphology. Figure 2A–I shows images for *F. oxysporum* (PV226192) while (J,L) shows the images for *F. oxysporum* (PV247065). Images (P–R) demonstrate the morphological changes in *A. alternata*. Figure 2A,G,G illustrates the structure of *F. oxysporum* (PV226192) under normal growth conditions. From the SEM image (A) at magnification of 2500×(scale range 50 μm), it is evident that the growth and surface of the fungi appear to be dense and intact as compared to the growth under treatment conditions (MCF 20 mg/mL). Hyphal width measured by imageJ software varied between 0.53 to 0.57 μm. The hyphae appear to be shrunk and deformed for fungi treated with MCF 20 mg/mL with hyphal width estimating 0.43, 0.34, and 1.19 μm showing no consistency in structure (Figure 2B,C). In some regions, the hyphae are very thin (B) while at other regions, they are broadened (C). The broadened hypha measures about 3.06 μm. The same phenomenon was observed under a light microscope as shown in Figure 1D. The control is shown at a higher magnification (5000×) in Figure 2D where the surface appears to be smooth with no obvious depressions and varying hyphal widths, ranging approximately between 0.68 μm to 0.85 μm. Contrary to it, Figure 2E,F shows apparent distorted structures or hollow regions at the same magnification (marked red). The smaller hollow region has width of approximately 0.73 μm and height of 0.48 μm while the larger hollow region has a width of about 0.76 μm and height of 0.91 μm. Control image g in Figure 2 at 10,000× magnification measures hyphal width to be in range of 1.23 to 1.66 μm. The walls of fungi shown in Figure 2H appears to have hollow spaces (h), one indicated by red arrow, with an estimated width of 2.61 μm and height of 1.06 μm. Compacted hyphal walls (Figure 2I) are observed, when treated with ET at concentration of 20 mg/mL. The compaction appears to have spherical structures with approximate widths ranging between 1.27 to 1.05 μm while heights ranging between 1.85 to 1.64 μm. Similar compaction was observed under a light microscope as indicated in Figure 1F. The spores of *F. oxysporum* (PV247065) in control (Figure 2J) and treatment have distinct differences (Figure 2K,L). The spores in control group appear to be round with smooth surface. Area, roundness, circularity, and solidity were automatically measured by imageJ software. The smaller spore has an approximate area of 1.363 μm^2^ with an aspect ratio (AR) of 1.038. Its roundness, circularity, and solidity values were estimated to be 0.963, 0.960, and 0.898 respectively. The larger spore had an area of roughly 4.054 μm^2^ and an AR of 1.279. Its roundness, solidity, and circularity values were nearly 0.782, 0.965, and 0.885. The values indicate that spores in control group are round with values close to 1, circular, and have smooth compact structures. Contrary to control group, spore observed in Figure 2K has a rough surface with area of about 22.45 μm^2^, AR of 1.264, roundness of 0.791, solidity of 0.973, and circularity of 0.850. Treatment group with larger spore area compared with control indicates possible deposition of compounds from extract over the spore surface leading to potential deformation or bursting. Moreover, spores in Figure 2K appeared to be broken (labelled 1) with circularity of about 0.573, a cavity (labelled 2) with circularity of 0.541, and had punctures (labelled 3). *A. alternata* SEM images showed noticeable variations in the structures of both mycelia and spores. Figure 2M,P show intact hypha (marked red) and a demarcated spore (area: 125.791 μm^2^) with relatively preserved surface structures. Figure 2N shows the damaged or broken hyphae at two regions (marked red) when treated with ET extract at 20 mg/mL. Figure 2O indicates the rough and shrunk surface of the fungi. Figure 2Q (indicated by arrow) shows pronounced structural damage where the external cover of the spore (approximate area: 90.916) structure is completely broken off. Furthermore, the structure of the spore appears to be porous and distorted (marked red) in Figure 2Q,R treated with MCF extract at a concentration of 20 mg/mL. The area covered by spore in treated group is much less than control group demonstrating the effect of extract on spore growth and morphological patterns.

Other research reported comparable SEM micrographs of *F. oxysporum* species. One of the studies reported the smooth structure of mycelia when grown under normal conditions. However, when treated with copper oxide nanoparticles (CuONPs), the mycelia happened to have a rough surface with severe distortion, dehydration, and shrinkage (El‐Sayed et al. 2023). Similar morphological alterations in the mycelia of *A. alternata* are reported with the organic extract of a herb, *Artemisia sieberi* (Al Otibi and Rizwana 2019). Similarly, in another research, the effect of fruit *Annona muricata* L. extracts exhibited similar structural deformations in *A. alternata* (Rizwana et al. 2021).

### 3.4 Fruit Bioassays to Assess the Potential of the Extract as a Biocontroller Against *F. oxysporum* (PV226192) in Cucumber

**FIGURE 3:**
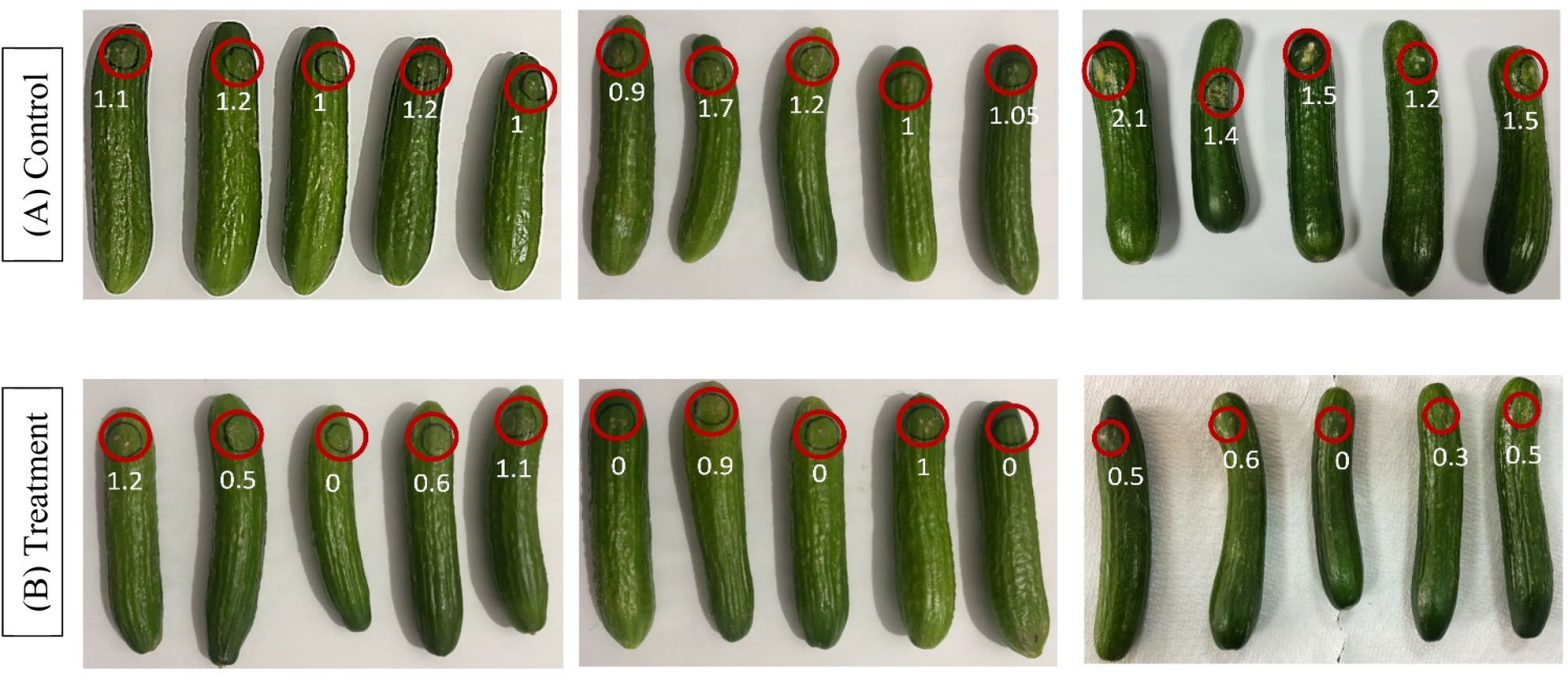
Study of curative effects of extract on cucumber. (A) Control group; cucumbers inoculated with *F. oxysporum* (PV226192) and sterile distilled water twice without the application of extract. (B) Treatment group: Cucumbers inoculated with *F. oxysporum* (PV226192) and sprayed twice with ethanolic (ET) extract at concentration of 5 mg/mL. Red circles indicate the wounds. Numbers on the cucumber demonstrate the measurement of growth of fungi in cm.

Based on the results obtained from the in vitro investigation of antifungal activity of the extracts, a preliminary experiment was conducted to inspect how the extract behaves when in contact with fungi and fruit at the same time (Figure 3). *F. oxysporum* was chosen for the in vivo assay due to its aggressive nature, rapid fruit infection capability, and relevance to crop diseases in arid regions. Though the extract suppressed the growth of the fungi in vitro through morphological alterations, a significant difference was seen between cucumbers in both groups. Based on two‐tailed *t*‐test statistical analysis, treatment group clearly indicated a significant (*t* = 3.103, *p* = 0.036) reduction in diameter of lesion as compared with control group. Moreover, using Equations (3, 4, and 5), further calculations were made. The average disease incidence (DI) of replicates was 100% for the control group while 66.7% for the treatment group. Moreover, the average disease severity (DS) of replicates of treatment group was 37.8%. The extract efficacy (EE) is 62.2% which correlates to the in vitro results. This experiment was the first to evaluate the in vivo behaviour of our extracts. Although the absence of positive control is a limitation of the study, the satisfactory results obtained provide a basis for future large‐scale experiments, which will include a commercial fungicide as a positive control. The use of natural compounds as biocontrol agents will reduce human reliance on fungicides, ultimately reducing environmental and biological hazards.

Fruits and vegetables, upon contact/invasion with pathogens, tend to release stress defence signalling pathways mediated by hormones (Peng et al. 2018). The natural defence mechanism, with the addition of extract, helps in fighting against the pathogen. From our study, we can report that the natural mechanism, along with the macroalgal extract, reduced the growth of fungi, thus reducing the spoilage of cucumbers. However, there is a possibility to completely inhibit the fungal growth by manipulating the extract through the addition of a secondary biocontrol agent.

### 3.5 GC/MS Analysis of Extracts

The GC/MS spectrum identified peaks for different compounds as described in Table 3. For the ET extract, 14 compounds were identified, of which multiple compounds were listed in the NIST 20 library for a peak at a residence time of 9.920 min. For the MCF extract, 6 compounds were identified.

**TABLE 3:** GC/MS analysis of ethanolic and methanol: Chloroform extracts with the Residence time (min), Scoring index (SI), name of compounds, chemical formula, and molecular weight (g/mol).

Residence time (min)	SI	Compound name	Chemical formula	Molecular weight (g/mol)
Ethanolic extract				
1.395	93	Propylene Glycol	C_3_H_8_O_2_	76
		(S)‐(+)‐1,2‐Propanediol		
9.755	91	n‐Hexadecanoic acid	C_16_H_32_O_2_	256
9.920	79	9‐Tricosene	C_23_H_46_	322
9.920	79	Disparlure (2‐Nonadecanone)	C_19_H_38_O_0_	282
9.920	79	1‐Tetracosene	C_24_H_48_	336
9.920	79	1‐Eicosanol	C_20_H_42_O	298
9.920	79	1‐Hexacosene	C_26_H_52_	364
10.890	85	Oleic Acid	C_18_H_34_O_2_	282
11.055	85	Ethyl Oleate	C_20_H_38_O_2_	310
12.070	89	8,11,14‐Eicosatrienoic acid	C_20_H_34_O_2_	306
		9,12,15‐Octadecatrienoic acid		
14.750	90	6,10,14,18,22‐Tetracosapentaen‐2‐ol	C_30_H_51_BrO	506
20.540	81	Ergosta‐5,24(28)‐dien‐3‐ol	C_28_H_46_O	398
Methanol: Chloroform extract				
1.265	93	Trichloromethane	ChCl_3_	118
9.525	65	Octadecanoic acid	C_33_H_66_O_3_	510
10.610	70	6,9‐Octadecadienoic acid	C_19_H_34_O_2_	294
10.805	94	Methyl Stearate	C_19_H_38_O_2_	29
11.635	94	Docosapentaenoic Acid methyl ester	C_23_H_36_O_2_	344
14.760	77	Lupeol	C_30_H_50_O	426

The GC–MS spectrum revealed the presence of different compounds, with most of them having antimicrobial and other biological properties. Some of the peaks correspond to compounds 1, 2‐propanediol, n‐hexadecanoic acid, eicosanol, oleic acid, octadecatrienoic acid, docosapentaenoic acid (DPA) methyl ester, and Lupeol (Table 3).

Compound 1,2‐Propanediol in ET extract has antimicrobial (both antibacterial and antifungal) properties (Nalawade et al. 2015). Generally, diols are reported to exhibit antimicrobial activities depending on the aliphatic chain length and the carbon numbers on which the hydroxyl (–OH) groups are present (Yoo et al. 2015). Another compound, n‐hexadecanoic acid found in the GC/MS spectrum is a fatty acid ester (Akwu et al. 2019) and also exhibits antimicrobial properties (Manilal et al. 2009). Multiple pathways are reported through which n‐hexadecanoic acid inhibits fungal growth. Being a long‐chain fatty acid, n‐hexadecanoic acid integrates into fungal membranes, leading to destabilisation of the lipid bilayer and disruption of the membrane's integrity. This destabilisation further neutralises processes of enzymes incorporated within the membranes, most importantly phospholipase A2 (PLA2). This enzyme plays a major role in the biosynthesis of significant membrane lipids like triacylglycerol and sphingolipid (Guimarães and Venâncio 2022; Ghfil et al. 2025). Moreover, nutrient uptake mechanisms are impaired as well. As a result of stress, the production of reactive oxygen species (ROS) is induced that damages DNA, proteins, and other related activities. Another long‐chain fatty acid, 1‐eicosanol, is also known for its antimicrobial properties (Chatterjee et al. 2018). Similarly, oleic acid, which is an unsaturated fatty acid, and its derivative ethyl oleate (Yasari et al. 2021) are also reported to have antifungal activity (Guimarães and Venâncio 2022). The GC/MS analysis revealed the presence of trienoic fatty acids (TFA/TUFA) in the ET extract. TFA/TUFA is the type of polyunsaturated fatty acid that does not auto‐oxidise abruptly as compared to other types and is less prone to oxidation reactions over time (Roberts and Durand 2004). Such fatty acids are involved in the biosynthetic pathways by being precursors of unique compounds like oxylipins that have antifungal potentialities (Guimarães and Venâncio 2022; Barbosa et al. 2016). The compounds present in the ET extract are attributed to the polarity of the solvent used. ET solvent can solubilise amphiphilic compounds due to the presence of a polar carboxyl group on one side, for instance, free fatty acids. Due to the polar nature of ET, fatty acids and their derivatives are mainly favoured, hence dominant in the GC/MS spectrum. For the spectrum of MCF extract, peaks of different compounds were seen with unique properties such as octadecanoic acid (stearic acid) and methyl stearate. These compounds are known for their antimicrobial properties (Rahman et al. 2014; Sharaf et al. 2022). Octadecanoic acid is a saturated fatty acid targeting mainly the fungal membrane. The acid's mode of action is by insertion into the fungal membrane causing membrane disruption, fluidity imbalance, and leakage of intracellular components, eventually leading to fungal death. Another important feature of saturated fatty acids is their hydrophobicity, which enhances their role as bioactive compounds (Mugayi and Mukanganyama 2024). Furthermore, a peak for docosapentaenoic acid (DPA) methyl ester, a long‐chain fatty acid, is found in the GC/MS spectrum of MCF. DPA is a well‐known fatty acid reported in literature for its unique bioactive properties (Kaur et al. 2016). Lastly, lupeol from the family of terpenoids with antimicrobial properties (Liu et al. 2021) was found to be present in the MCF extract, as confirmed by the GC/MS spectrum. Lupeol is a pentacyclic triterpenoid, belonging to the phytosterol family (Luo et al. 2025). It is a very well‐known compound for its versatile functionalities. But its antimicrobial mechanism of action is not studied in detail. A study reported its fungistatic activity against *Candida parapsilosis* . They found that lupeol interacts with a key fungal enzyme called sterol 14α‐demethylase (CYP51). This enzyme plays a vital role in the biosynthesis of ergosterol. The study reveals through molecular docking that the compound lupeol binds at the same ligand that is generally targeted by fungicides such as azoles. This phenomenon impairs ergosterol synthesis, leading to the accumulation of toxic sterol, leading to cell death (da Silva Dutra et al. 2025). Compounds found in the GC/MS spectrum of MCF extract reflect the characteristics of MCF solvent, a combination of polar and non‐polar phases, hence effective at extraction of both hydrophobic and hydrophilic compounds. For instance, chloroform allows the solubilisation of fatty acids and triterpenoids, whereas methanol assists in disruption of cells and release of lipophilic components. The presence of peaks corresponding to fatty acids and other compounds in the GC/MS spectrum, which have been reported to possess antimicrobial properties, may indicate their possible involvement in the antifungal activity of the tested extract. Although the direct mechanism of antifungal action was not investigated in the present study, it is hypothesised that these bioactive compounds could interact with fungal cells, potentially leading to the disrupted hyphal morphologies observed by SEM.

Based on literature, it is clear that brown macroalgae is home to fatty acids found in varying types with different saturation indices; saturated fatty acids (SFA), monounsaturated fatty acids (MUFA), and polyunsaturated fatty acids (PUFA) (Mohamed and Saber 2019). Previously, a research conducted in Qatar investigated 12 algae for their composition. It was reported that algae *Hormophysa cuneiformis* (the now *Hormphysa triquetra*) had different fatty acids. Some compounds listed are oleic acid, arachidonic acid, linolenic acid, and eicosadienoic acid (Heiba et al. 1997). Further, another research utilising *H. trqieutra* against bacteria revealed the presence of compounds such as palmitic acid, oleic acid, stearic acid, and decanoic acids (El Shoubaky and Salem 2014).

Seaweeds are home to many valuable compounds, such as palmitic acid, myristic acid, terpenes, phenols, and phlorotannins (Akbar and Mustari 2024; Balamurugan et al. 2013; Samarakoon and Jeon 2012). Similar compounds, such as oleic acid, hexadecanoic acid, and octadecanoic acid were isolated from macroalga *H. musciformis* ethanolic extract (Balamurugan et al. 2013). Similarly, the methanolic extract of *P. pavonica* revealed the presence of a similar compound to oleic acid (Usha et al. 2015). The methanolic extract of *Ulva lactuca* identified similar compounds in the GC/MS spectrum that include palmitic acid and oleic acid, whereas the methanolic extract of *U. fasciata* indicated the presence of octadecenoic acid, methyl ester, and octadecadienoic acid, methyl ester apart from palmitic acid (Shobier et al. 2016). The presence of compounds with antimicrobial potentialities might indicate their involvement in combating the pathogenic fungi either as a group of compounds or alone. Our study confirmed that the majority of compounds are from the group of fatty acids. Fatty acids have several mechanisms through which they inhibit the growth of fungi. The first and foremost mechanism is the integration of fatty acids into the lipid membrane of fungi, leading to compromised membrane integrity and loss of components through its deformed structure (Avis and Bélanger 2001). In another mechanism, fatty acids alter the unsaturation ratio of the fungal membrane, due to which reactive oxygen species (ROS) start to accumulate, leading to loss in the mitochondrial membrane potential (Thibane et al. 2012). Lastly, fatty acids can also affect the DNA mechanisms, such as the repair, replication, and so on by inhibiting important enzymes (Pommier 2006; Yonezawa et al. 2005).

Despite the promising antifungal activity of crude extracts of macroalga observed, the current study has certain limitations and concerns that should be considered and acknowledged. Both molecular and biochemical approaches should be employed in future studies to further evaluate the mechanism of action of crude extracts against fungi. Moreover, the number of phytopathogens under investigation should be expanded so as to cover a wide spectrum, thereby validating applicability of macroalgal extracts against fungal pathogens. Additionaly, in vivo experiment conducted was preliminary; thus, a large‐scale experiment under controlled and field conditions are mandatory to assess in details the efficacy, toxicity, and environmental safety of macroalgal extracts under natural conditions. It is important to mention here that our study was limited to in vitro screening of antifungal activities and preliminary in vivo investigation of extract efficacy in cucumbers. The study did no shed a light on the toxicity of extracts and non‐target microbial selectivity testing. Based on the results of this study, it is recommended for future studies to consider the phytotoxicity screening (seed germination/seedling growth), and toxicity tests against reported beneficial phyto‐microbes. Considering the environmental aspects of using macroalgae‐based biofetilizers, it is generally regarded ecologically safe due to being composed majorily of organic compounds (Ali et al. 2021).

## 4 Conclusion

In this study, we assessed the antifungal activities of the brown macroalga *Hormophysa triquetra* by preparing extracts with three solvents: aqueous, ethanol, and methanol: chloroform. The aqueous extract did not show antifungal activity against any tested fungi. However, the ethanolic and methanol: chloroform extracts were effective against *F. oxysporum* (PV226192), *F. oxysporum* (PV247065), and *A. alternata*. The microscopic images showed morphological alterations in fungi when treated with extracts that were further confirmed by SEM analysis. In vivo analysis showed that the extract is able to limit the fungal infection in cucumber as compared to the control group with an extract efficiency of 66.7%. GC/MS analysis identified several antifungal compounds that disrupt the fungal cell walls, altering their morphology.

Although these results demonstrate promising antifungal activity of crude *H. triquetra* extracts, they represent preliminary evidence. Further investigations are required to explicate the precise mode of action, expanding spectrum of phytopathogens, and evaluation of phytotoxicity and long‐term environmental safety of the extracts usage under both greenhouse and field conditions. Despite these limitations, the current study provides primary insights into the potential use of macroalgal extracts as a sustainable approach that could contribute to decreasing the excessive use of chemical pesticides following further validation. Additionally, previous research suggests that macroalgae possess antifungal properties (Ammar et al. 2022; Raghunandan et al. 2019), and may be explored for agricultural applications, such as fertilisers, and as biocontrol agents to help mitigate post‐harvest decay in fruits and vegetables.

## Author Contributions

**Shazia Bibi:** conceptualization, methodology, writing – original draft preparation, investigation, formal analysis, data curation, validation. **Samir Jaoua:** validation, writing – reviewing and editing. **Nabil Zouari:** validation, writing – reviewing and editing. **Mohammad A. Al‐Ghouti:** validation, writing – reviewing and editing. **Mohammed H. Abu‐Dieyeh:** conceptualization, validation, supervision, resources, methodology, writing – reviewing and editing.

## Conflicts of Interest

The authors declare no conflicts of interest.

## Data Availability

The data that support the findings of this study are available from the corresponding author upon reasonable request.
